# *APOE4* is associated with cognitive and pathological heterogeneity in patients with Alzheimer’s disease: a systematic review

**DOI:** 10.1186/s13195-020-00712-4

**Published:** 2020-11-04

**Authors:** Sheina Emrani, Hirra A. Arain, Cassandra DeMarshall, Tal Nuriel

**Affiliations:** 1 Department of Psychology, Rowan University, 201 Mullica Hill Road, Glassboro, NJ 08028 USA; 2 Department of Pathology and Cell Biology, Columbia University, 630 West 168th Street, New York, NY 10032 USA; 3 Taub Institute for Research on Alzheimer’s Disease and the Aging Brain, Columbia University, 630 West 168th Street, New York, NY 10032 USA; 4 Department of Geriatrics and Gerontology, Rowan University School of Osteopathic Medicine, One Medical Center Drive, Stratford, NJ 08084 USA

**Keywords:** Apolipoprotein E, APOE, APOE4, Alzheimer’s disease, AD, Heterogeneity

## Abstract

Possession of the ε4 allele of apolipoprotein E (*APOE*) is the primary genetic risk factor for the sporadic form of Alzheimer’s disease (AD). While researchers have extensively characterized the impact that *APOE* ε4 (*APOE4*) has on the susceptibility of AD, far fewer studies have investigated the phenotypic differences of patients with AD who are *APOE4* carriers vs. those who are non-carriers. In order to understand these differences, we performed a qualitative systematic literature review of the reported cognitive and pathological differences between *APOE4*-positive (*APOE4*+) vs. *APOE4*-negative (*APOE4*−) AD patients. The studies performed on this topic to date suggest that *APOE4* is not only an important mediator of AD susceptibility, but that it likely confers specific phenotypic heterogeneity in AD presentation, as well. Specifically, *APOE4*+ AD patients appear to possess more tau accumulation and brain atrophy in the medial temporal lobe, resulting in greater memory impairment, compared to *APOE4*− AD patients. On the other hand, *APOE4*− AD patients appear to possess more tau accumulation and brain atrophy in the frontal and parietal lobes, resulting in greater impairment in executive function, visuospatial abilities, and language, compared to *APOE4*+ AD patients. Although more work is necessary to validate and interrogate these findings, these initial observations of pathological and cognitive heterogeneity between *APOE4*+ vs. *APOE4*− AD patients suggest that there is a fundamental divergence in AD manifestation related to *APOE* genotype, which may have important implications in regard to the therapeutic treatment of these two patient populations.

## Introduction

### *APOE4* carriers have an increased risk of developing AD

In 1993, Roses and colleagues first reported that an individual’s risk of developing Alzheimer’s disease (AD) is increased if they carry the ε4 allele of apolipoprotein E (*APOE*) [1–3], an important apolipoprotein that had primarily been studied for its role in transporting cholesterol and other lipids through the periphery and within the brain [4–6]. Since that time, the link between *APOE4* and AD susceptibility has been extensively validated and characterized. A 1997 meta-analysis by Farrer et al. nicely summarizes the general associations between *APOE* genotype and AD susceptibility [7], which has remained relatively consistent in future studies [8–10]. For example, while the *APOE2*, *APOE3*, and *APOE4* alleles are present in cognitively normal Caucasians at a relative frequency of about 8%, 78%, and 14%, respectively, *APOE4* has an allele frequency of about 37% in Caucasian AD patients [7]. When broken down by the specific genotype frequencies, *APOE3/4* individuals represent about 21% of the cognitively normal Caucasian population, vs. about 41% of Caucasian AD patients (odds ratio [OR] 3.2), whereas *APOE4/4* individuals have a genotype frequency of about 2% in the cognitively normal Caucasian population, vs. about 15% in the AD-affected Caucasian population (OR 14.9) [7]. Furthermore, while possession of the *APOE2* allele is protective against AD [2, 10, 11], with Caucasian individuals who possess either the *APOE2/2* or the *APOE2/3* genotype having an OR of 0.6, this protective effect is overtaken by the risk effect of the *APOE4* allele in *APOE2/4* individuals (OR 2.6) [7].

These numbers shift, however, when the *APOE4*-associated risk of AD is stratified by traits such as age, gender, and ancestry. For example, the effects of the *APOE4* allele on AD risk are greatest in younger individuals, with the risk of AD among Caucasian *APOE3/4* individuals peaking at age 65 (OR ~ 4) and the risk of AD among Caucasian *APOE4/4* individuals peaking at age 60 (OR ~ 15.5) [7]. In terms of gender, numerous studies have found that the effects of *APOE4* on AD susceptibility are greater in women than in men [7, 12, 13], although these gender differences appear to decrease after age 75 [7, 8, 14]. For example, in the Farrer et al. meta-analysis, the authors reported that a 65-year-old Caucasian woman with an *APOE3/4* genotype has an OR of developing AD of over 4, whereas a 65-year-old Caucasian man with the same genotype has an OR of less than 2 [7]. Perhaps the most intriguing differences in *APOE4*’s effect size, however, are seen in individuals with different ancestral backgrounds. For example, individuals from African-ancestry populations, such as African Americans, have a higher general frequency of *APOE4* (*APOE4* allele frequency ~ 19%) than Caucasian populations, but these individuals are at a relatively lower risk of developing AD (*APOE3/4* OR 1.1; *APOE4/4* OR 5.7) [7]. However, the opposite appears to be true for East-Asian populations; for example, individuals of Japanese ancestry have a relatively low *APOE4* allele frequency (~ 9%), but a relatively high *APOE4*-related risk (*APOE3/4* OR 5.6; *APOE4/4* OR 33.1) [7].

**Fig. 1:**
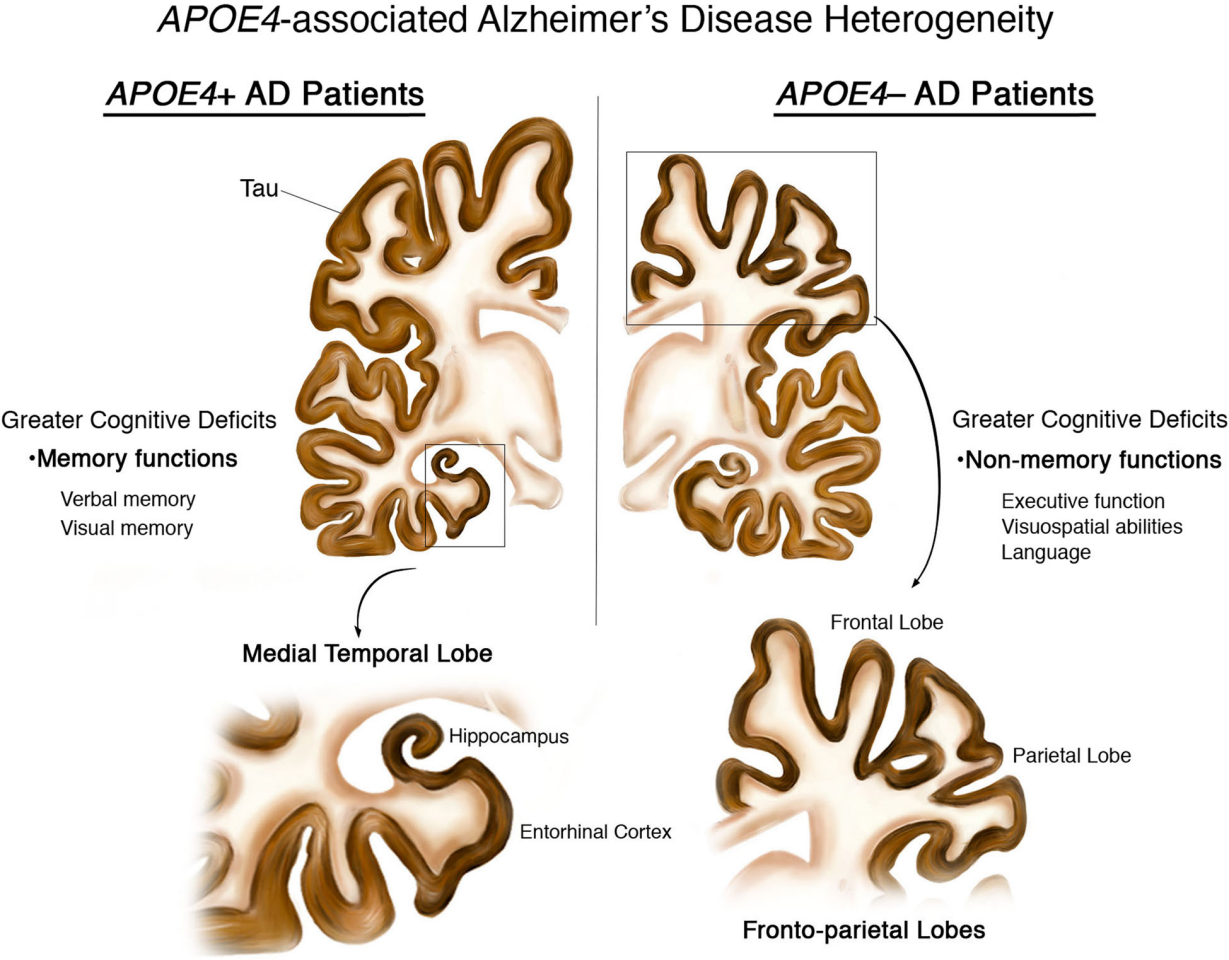
Cognitive and pathological heterogeneity in *APOE4+* vs. *APOE4−* AD patients. A representation of the heterogeneity reported in *APOE4+* vs. *APOE4−* AD patients. *APOE4*+ AD patients possess relatively more tau accumulation and brain atrophy in their medial temporal lobe, resulting in greater memory impairment, compared to *APOE4−* AD patients. On the other hand, *APOE4−* AD patients possess relatively more tau accumulation and brain atrophy in their fronto-parietal lobes, resulting in greater impairment in executive function, visuospatial abilities, and language, compared to *APOE4*+ AD patients. The level of tau accumulation (brown) represents the levels observed in AD brains during Braak stages V–VI

It is clear from these studies that the *APOE4* allele is a strong genetic risk factor for developing AD, even though the disease penetrance varies greatly with regard to age, gender, and ancestry. However, while extensive studies have characterized the role of *APOE4* in conferring AD risk, far fewer studies have investigated the effects of *APOE4* on the cognitive and pathological manifestation of the disease in individuals who have already converted to AD. In order to understand how a patient’s *APOE* genotype affects their disease presentation, we have performed a qualitative systematic literature review of the human studies that have been published to date examining the cognitive and pathological differences between *APOE4*-positive (*APOE4*+) vs. *APOE4*-negative (*APOE4*−) AD patients. Interestingly, these studies suggest that possession of *APOE4* does in fact result in phenotypic differences between *APOE4*+ vs. *APOE4−* AD patients, with *APOE4*+ AD patients appearing to possess relatively more tau accumulation and brain atrophy in the medial temporal lobe, resulting in greater memory impairment, than *APOE4*− AD patients, while *APOE4*− AD patients appear to possess relatively more fronto-parietal lobe tau accumulation and brain atrophy, resulting in greater impairment in executive function, visuospatial abilities, and language, than *APOE4*+ AD patients (Fig. 1).

## Methods

The data and information utilized in this qualitative systematic review were obtained from literature published between January 1, 1993, and June 1, 2020. A literature search using both electronic and manual search components was performed, with the goal of identifying all studies published during this time period that specifically compared AD presentation in *APOE4* carriers vs. *APOE4* non-carriers who were diagnosed with AD using standard methods. In order to accomplish this, PubMed was exhaustively searched to help identify articles containing a combination of keywords: *Apoliprotein E*, *APOE*, *APOE4*, *APOE4-positive*, *APOE4-negative*, *Alzheimer’s disease*, *AD*, and *patients*, which was followed by a first level screening of the articles’ titles and abstracts to identify studies that directly investigated our review topic. These searches were limited to studies with human subjects that were published in the English language. A manual reference check of the bibliographies of the relevant studies was also performed in order to identify additional articles that were not identified by the electronic search.

The full article of each identified study on this topic was downloaded and stored in a single folder, at which time a second level of screening of the full text was performed to confirm that each article directly compared the cognitive and/or pathological characteristics of *APOE4*+ vs. *APOE4−* AD patients. Lastly, each study was sorted into one or more of the following diagnostic categories: rate of cognitive decline (17 studies), neuropsychological profile (12 studies), brain atrophy (13 studies), Aß pathology (7 studies), or tau pathology (6 studies). Those studies that did not fit into one of these five categories were not included in the primary review.

We did not exclude studies based on any patient demographic characteristics or any specific methodologies employed. This broad inclusion criterion was utilized in order to provide the scientific community with a comprehensive record of the studies that have investigated the cognitive and pathological differences between *APOE4*+ vs. *APOE4−* AD patients to date. However, the demographic and methodological differences between studies were carefully considered in our overall conclusions, as is discussed throughout the review.

## Results

### *APOE4*+ AD patients do not appear to differ in their overall rates of cognitive decline compared to *APOE4−* AD patients

Although the heterogeneity between *APOE4*+ vs. *APOE4−* AD patients is an understudied phenomenon, one question that has been repeatedly investigated over the years is whether or not *APOE4*+ AD patients undergo an accelerated rate of cognitive decline as compared to *APOE4−* AD patients. However, the results of these studies have been decidedly mixed. While numerous groups have reported that *APOE4*+ AD patients do in fact experience a more accelerated cognitive decline compared to *APOE4−* AD patients [15–20], other studies have shown either no *APOE* genotype-associated differences in the rate of cognitive decline in AD patients [21–27] or slower cognitive decline in *APOE4*+ vs. *APOE4−* AD patients [28–30] (Table 1).

**Table 1:** Studies investigating the effects of *APOE4* on the rate of cognitive decline in AD patients

Study	Study details	Participant details	Study results
***APOE4*** **associated with accelerated cognitive decline**			
Cosentino et al. [15]	570 AD patients (WHICAP and Predictors Study cohorts) were recruited and followed for an average of 4 years. Outcome variable was a composite cognitive *z*-score from five cognitive domains (memory, abstract reasoning, visuospatial, language, and executive speed).	Mean age for two population-based cohorts (WHICAP) and one clinic-based cohort (Predictors Study) participants was 81.97 (*n* = 199; 73% female, 61% Hispanic, 31% African American), 80.70 (*n* = 215; 76% female, 62% Hispanic, 28% African American), and 75.30 (*n* = 156; 58% female, 0% Hispanic, 5% African American) years of age, respectively.	The effect of *APOE4* on rate of cognitive decline varied across samples. *APOE4*+ AD patients in the incident sample demonstrated an accelerated rate of cognitive decline compared to *APOE4*− AD patients. Caucasian participants were more likely to show an association between *APOE4* status and rate of cognitive decline as compared to Hispanic and African American participants.
Martins et al. [16]	218 AD patients (OPTIMA cohort) were evaluated for cognitive ability using the Cambridge Examination for Mental Disorders of the Elderly (CAMDEX) scale.	Mean age for *APOE4/4* was 71.1 (*n* = 28, 55% female), *APOE3/4* was 73 (*n* = 97; 54% female), *APOE2/4* was 75.8 (*n* = 8; 75% female), *APOE3/3* was 75.2 (*n* = 69; 55% female), *APOE2/3* was 74.2 (*n* = 15; 53% female), and *APOE2/2* was 79.5 (*n* = 1; 100% female). No ancestry information was provided.	*APOE4*+ AD patients demonstrated both an earlier and faster rate of cognitive decline compared to *APOE4*− AD patients. *APOE4/4* patients progressed faster than *APOE3/4* patients.
Craft et al. [17]	201 probable AD patients were evaluated using the Dementia Rating Scale (DRS) and followed for 1–6 years to measure the rate of cognitive decline.	Mean age for *APOE4/4* was 74.0 (*n* = 30; 77% female), *APOE3/4* was 78.6 (*n* = 82; 59% female), *APOE3/3* was 79.8 (*n* = 75; 60% female), and *APOE2/3* was 77.3 (*n* = 14; 36% female). No ancestry information was provided.	*APOE4*/*4* AD patients demonstrated an accelerated rate of cognitive decline compared to *APOE4*− AD patients.
Hirono et al. [18]	64 AD patients were evaluated using the ADAS-Cog and followed for 1 year to measure the rate of cognitive decline.	Mean age for *APOE4*+ homozygotes was 70.8 (*n* = 8; 25% female), *APOE4*+ heterozygotes was 73.6 (*n* = 33; 83% female), and *APOE4*− was 76.3 (*n* = 23; 74% female). No ancestry information was provided.	*APOE4*+ AD patients demonstrated an accelerated rate of amnestic (assessed by word recall and recognition subtests) and overall cognitive decline, which was significantly correlated with the number of *APOE4* alleles.
Kanai et al. [19]	33 AD patients were evaluated with the Mini-Mental State Examination (MMSE) and CSF biomarkers, and followed for up to 20 months to measure the rate of cognitive decline.	Mean age for both *APOE4*+ (*n* = 17; 59% female) and *APOE4*− (*n* = 16; 69% female) AD patients was 65. No ancestry information was provided.	*APOE4*+ AD patients demonstrated a more rapid decrease in MMSE score, as well as increased levels of CSF tau compared to *APOE4*− AD patients.
Chang et al. [20]	104 AD patients (ADNI cohort) and 123 controls were evaluated for neuropsychological and morphometric changes stratified by age (young-old vs. very-old) and *APOE4* status.	Mean age of young-old *APOE4*+ AD patients was 70.84 (*n* = 49; 57% female), very-old *APOE4*+ AD patients was 83.70 (*n* = 20; 25% female), young-old *APOE4*− AD patients was 70.53 (*n* = 15; 53% female), and very-old *APOE4*− AD patients was 84.16 (*n* = 20; 60% female). No ancestry information was provided.	Young-old (≤ 75 years old) *APOE4*+ AD patients demonstrated greater cognitive decline in memory and language over a 1-year interval as compared to other groups, suggesting that the effect of *APOE* status on rate of decline is dependent upon age at onset of disease.
**No association between cognitive decline and** ***APOE4*** **status**			
Kleiman et al. [21]	366 AD patients were evaluated with the MMSE, ADAS-Cog, and daily function scales (Instrumental Activities of Daily Living, IADL; Alzheimer’s Disease Cooperative Study-Activities of Daily Living, ADCS-ADL), and followed for up to 1.8 years to measure the rate of cognitive decline.	Mean age for *APOE4*+ homozygotes was 71.6 (*n* = 51; 70.6% female), *APOE4+* heterozygotes was 74.4 (*n* = 159; 59.1% female), and *APOE4*− was 73.4 (*n* = 156; 59.6% female). No ancestry information was provided.	*APOE4* status did not influence the rate of disease progression in either cognitive or functional domains of assessment, regardless of allele dose.
Growdon et al. [22]	66 probable AD patients were evaluated using nine cognitive tests assessing explicit memory, attention, language, visuospatial function, frontal-lobe function, and logical reasoning abilities for up to 5.5 years to measure the rate of cognitive decline.	56% of study participants were female. Mean age for *APOE4*+ homozygotes was 68.6 (*n* = 16), *APOE4*+ heterozygotes was 70.3 (*n* = 34), and *APOE4*− was 65.5 (*n* = 16). No ancestry information was provided.	*APOE4* status did not influence the rate of cognitive decline across *APOE* genotypes.
Holmes et al. [23]	164 late-onset AD patients were evaluated for cognitive and non-cognitive abilities to measure the rate of cognitive decline.	Mean age for *APOE4*+ AD patients was 75.5 (*n* = 92) and *APOE4*− AD patients was 78.7 (*n* = 72). No ancestry information was provided.	*APOE4* status was found to be associated with an earlier age of onset, but was not found to influence the cognitive progression of the disease.
Kurz et al. [24]	64 AD patients were evaluated using the Cambridge Cognitive Examination (CAMCOG), the MMSE, and the Dementia Scale (DS) included in the CAMDEX, and followed for over 3 years to measure the rate of cognitive decline.	The study included 14 males and 50 females, with an average age of 73. No ancestry information was provided.	*APOE4* status was found to have no significant impact on the rate of deterioration in everyday performance, the rate of cognitive decline, or on baseline function and progression of the disease.
Basun et al. [25]	60 late-onset AD patients were evaluated using the MMSE over 3 years to measure the rate of cognitive decline.	Mean age for *APOE4*+ AD patients was 81.6 (*n* = 27; 85% female) and *APOE4*− AD patients was 85.8 (*n* = 33; 82% female). No ancestry information was provided.	*APOE4*+ AD patients demonstrated a significantly lower age at disease onset and longer duration, but no significant differences were found in the MMSE test scores over time between carriers and non-carriers.
Murphy et al. [31]	86 probable AD patients were evaluated using the Mini-Mental State Examination (MMSE) over an average of 3.6 years to measure the rate of cognitive decline.	Participant details not available.	No association between *APOE4* allele dosage and rate of cognitive decline was found.
Farlow et al. [26]	959 AD patients were treated with either metrifonate or placebo for a period of up to 26 weeks to measure the effects of treatment and *APOE4* status on cognitive decline using the ADAS-Cog and Clinician’s Interview-Based Impression of Change with Caregiver Input (CIBIC-Plus).	Mean age (placebo) for *APOE4*+ homozygotes was 70.5 (*n* = 59; 62.7% female, 69.5% Caucasian), *APOE4*+ heterozygotes was 74.1 (*n* = 183; 67.2% female, 72.1% Caucasian), and *APOE4*− was 73.2 (*n* = 132; 57.6% female, 73.5% Caucasian). Mean age (metrifonate) for *APOE4*+ homozygotes was 72.1 (*n* = 68; 57.4% female, 77.9% Caucasian), *APOE4*+ heterozygotes was 74.4 (*n* = 281; 67.3% female, 80.4% Caucasian), and *APOE4*− was 73.5 (*n* = 236; 61.4% female, 78.8% Caucasian).	*APOE4* genotype in conjunction with metrifonate treatment had no significant effect on global function and cognitive performance in AD patients. *APOE4* genotype was not found to influence the rate of disease progression in placebo-treated AD patients.
Aerssens et al. [27]	1528 probable AD patients were treated with either galantamine or sabeluzole or placebo for a period of up to 1 year to measure the effects of treatment and *APOE4* status on cognitive decline using the MMSE and ADAS-cog, along with the Disability Assessment for Dementia (DAD).	Mean age was 74.2 (59% female). No ancestry information was provided.	*APOE4/4* AD patients demonstrated a lower age of disease onset, but allele status did not influence the rate of cognitive decline (including in placebo-treated group), or the effectiveness of galantamine treatment.
***APOE4*** **associated with slower cognitive decline**			
Stern et al. [28]	99 probable AD patients (WHICAP cohort) were evaluated using a modified MMSE, as well as other cognitive and motor measures, and were followed biannually for up to 6 years to measure the rate of cognitive decline.	Mean age for *APOE4*+ homozygotes was 69.7 (*n* = 15; 66.7% female, 80% Caucasian, 20% African American), *APOE4+* heterozygotes was 71.8 (*n* = 41; 43.9% female, 82.9% Caucasian, 4.9% African American, and 12.2% Hispanic), and *APOE4*− was 71.3 (*n* = 43; 44.2% female, 100% Caucasian).	*APOE4*+ AD patients demonstrated a lower rate of mortality, slower rate of decline in MMSE scores, less brain atrophy, and a delayed development of myoclonus than *APOE4*− AD patients. The presence of *APOE4* allele was associated with an earlier age of onset of AD.
Frisoni et al. [29]	62 sporadic late-onset (≥ 70 years of age) AD patients were evaluated using the MMSE and Clinical Dementia Rating (CDR) to measure the relationship between disease progression and *APOE4* status.	Mean age of *APOE4*+ homozygotes was 78.6 (*n* = 19; 74% female), *APOE4*+ heterozygotes was 79.8 (*n* = 16; 81% female), and *APOE4*− was 80.7 (*n* = 27; 93% female). No ancestry information was provided.	*APOE4*+ AD patients demonstrated a longer disease duration compared to *APOE4*− AD patients.
Hoyt et al. [30]	189 probable AD patients were evaluated using individual growth curve analyses for up to 2 years to measure the rate of cognitive decline using various neuropsychological tests.	Mean age for *APOE4+* homozygotes was 69.2 (*n* = 22; 91% female), *APOE4+* heterozygotes was 72.7 (*n* = 82; 79% female) and *APOE4*− was 73.2 (*n* = 47; 68% female). No ancestry information was provided.	*APOE4/4* AD patients demonstrated a slower rate of decline on global cognitive functioning, but not for measures of specific cognitive functions.

In general, these discrepancies between the reported rates of cognitive decline in *APOE4*+ vs. *APOE4−* AD patients highlight the difficulty of trying to determine a consensus about the contributions of one single trait, such as *APOE* genotype, on the overall presentation of AD. Adding to this difficulty, each of the studies that we have cited in this review utilizes different methods for their analysis, and the patient populations that they assessed often vary widely in their demographic characteristics. As discussed in the “Introduction” section, differences in age, gender, and ancestral background are known to affect AD susceptibility among *APOE4* carriers; therefore, it is likely that these differences also affect AD presentation among *APOE4* carriers. Furthermore, in some of the studies we have cited, the authors have utilized a relatively small number of AD patients for their analysis; because of this, it is possible that type II statistical errors may affect the conclusions that these authors reported (i.e., a study's small sample size may have resulted in no differences being observed between *APOE* genotype groups, even if actual differences exist).

It should also be noted that a large number of the studies investigating the effects *APOE* genotype on AD presentation have focused on “probable” AD patients. Probable AD is classified using standardized cognitive screening tools and robust neuropsychological tests, and must follow a strict criteria, such as those described by the NINCDS-ADRDA workgroup in 1984 [32], or an updated criteria described by the NIA-AA workgroup in 2011 [33]. However, the utilization of cognitive profiles alone (or, likewise, the utilization of pathological markers alone) cannot give a 100% confident diagnosis of AD. Given this information, it is possible that some probable AD patients included in the studies cited in this review were misdiagnosed. Notably, it has been reported that *APOE4−* individuals make up the majority of AD-diagnosed patients who are later found to be Aß-negative by PET or at autopsy [34, 35]. For this reason, it is possible that the sole reliance on probable AD diagnosis in some of these studies could result in type I statistical errors that may affect their findings (i.e., a study's potential inclusion of non-AD patients, especially if this was weighted towards the *APOE4*− individuals, may have resulted in significant differences being observed between *APOE* genotype groups, even if none exist). Rather than excluding such studies, however, we chose to include them, but to take their limitations into account in our overall, qualitative assessment of the data.

In regard to *APOE4*’s effects on the rate of cognitive decline in AD, assessing the demographic and methodological differences between the studies listed above does provide some clarity. For example, in many of these studies, the authors had access to a relatively small number of AD patients. One potential approach to assess the findings, therefore, is to focus only on the studies with a relatively large number of participants. Interestingly, when we only include the studies that meet a conservative threshold of *n* > 100 AD patients, there are three studies that reported accelerated cognitive decline in *APOE4*+ vs. *APOE4−* AD patients [15–17], four that reported no difference [21, 23, 26, 27], and only one that reported slower cognitive decline in *APOE4*+ vs. *APOE4−* AD patients [30]. Importantly, in the three largest studies from this group, a study by Kleiman et al. that analyzed 366 patients with probable AD [21], a study by Farlow et al. that analyzed 374 placebo-treated AD clinical-trial participants [26], and a study by Aerssens et al. that analyzed 504 placebo-treated AD clinical-trial participants [27], the authors *did not* find any *APOE4*-associated differences in the rate of cognitive decline in AD. These studies suggest that, when analyzed in a broad fashion, AD patients who carry the *APOE4* allele do not appear to possess a more aggressive form of the disease.

However, more work is needed to determine if *APOE* genotype may have a significant effect on the rate of cognitive decline in specific subsets of AD patients, such as within a given age group or gender or ancestry. For example, two of the highly powered studies referenced above, by Cosentino et al. and Craft et al., reported significant *APOE4*-associated increases in the rates of cognitive decline when looking specifically at incident (i.e., newly diagnosed) AD cases [15, 17]. This suggests that *APOE4* may accelerate cognitive decline at the earliest stages of AD diagnosis, but that these effects may dissipate with increasing disease severity. This possibility would be in line with what occurs prior to AD diagnosis, where *APOE4* carriers show increased conversion from mild cognitive impairment (MCI) to AD compared to non-carriers [36–38]. Similarly, non-demented elderly *APOE4* carriers have also been reported to undergo increased cognitive decline compared to non-demented elderly non-carriers [39, 40], especially when these *APOE4* carriers are positive for Aß [41–43].

### *APOE4*+ AD patients have a more amnestic cognitive profile than *APOE4−* AD patients

Another factor that deserves critical attention is the multi-faceted nature of the cognitive presentation of AD. For example, AD patients are not only prone to the characteristic amnestic symptoms commonly associated with the disease; they are also prone to deficits in other cognitive domains, such as executive function, visuospatial abilities, and language [44]. Indeed, some atypical AD patients present with distinct non-amnestic cognitive phenotypes, including corticobasal syndrome (CBS), where patients present with movement impairment; frontal variant Alzheimer’s disease (fvAD), where patients present with behavioral/executive function impairment; logopenic variant primary progressive aphasia (lvPPA), where patients present with language impairment; and posterior cortical atrophy (PCA), where patients present with visual impairment. Furthermore, even within the overarching concept of memory, there is significant complexity that must be considered during the neuropsychological assessment of AD patients. For example, poor performance on immediate recall, delayed recall, and delayed recognition is typically suggestive of amnesia [45, 46]. However, difficulties on immediate and delayed recall, in the absence of reduced performance on delayed recognition, are suggestive of problems with lexical access, a task that is associated with significant frontal lobe involvement [47].

In order to assess whether *APOE* genotype may alter the cognitive profile of AD patients, a number of studies have utilized neuropsychological assessment tools—including cognitive screening tools, such as the Mini-Mental State Examination (MMSE); brief neuropsychological tests, such as the Alzheimer’s Disease Assessment Scale-Cognitive Subscale (ADAS-Cog); or more in-depth neuropsychological tests, such as the California Verbal Learning Test (CVLT)—in an attempt to parse out the potential divergence in cognitive deficits between *APOE4*+ vs. *APOE4−* AD patients [48–58]. Interestingly, the majority of these studies have reported that *APOE4*+ AD patients possess relatively more pronounced memory deficits than *APOE4−* AD patients [49–53, 58, 59], although a few studies did not find an association between *APOE* genotype and memory function [54, 55, 57]. In addition, a number of these studies have also reported that *APOE4−* AD patients possess relatively more pronounced deficits in non-memory cognitive domains, such as executive function, visuospatial abilities, and language, than *APOE4+* AD patients [48, 51–58], with a greater effect observed in younger *APOE4−* vs. *APOE4*+ AD patients [54, 58] (Table 2).

**Table 2:** Studies investigating the effects of *APOE4* on cognitive profiles in AD patients

Study	Study details	Participant details	Study results
***APOE4*** **associated with more pronounced memory deficits (only)**			
Marra et al. [49]	30 early-onset (< 65 years old) and 41 late-onset (> 70 years old) AD patients were evaluated for the effects of *APOE4* on the age at disease onset.	Mean age of early-onset *APOE4*+ AD patients was 58.8 (*n* = 20), early-onset *APOE4*− AD patients was 56 (*n* = 10), late-onset *APOE4*+ AD patients was 74.8 (*n* = 25), and late-onset *APOE4*− AD patients was 76.2 (*n* = 16). No gender or ancestry information was provided.	*APOE4*+ early-onset AD patients exhibited worse performance in measures of learning, long-term verbal memory, and general intelligence tasks. *APOE4* status had no effect on cognitive impairment at onset in late-onset AD patients.
Snowden et al. [50]	523 AD patients were evaluated to explore the relationship between *APOE* status and family history.	Mean age of 60 (56% female). No ancestry information was provided.	*APOE4*+ AD patients had an older age of onset, a positive family history, and demonstrated greater amnestic deficits than *APOE4*− AD patients. In contrast, frontal lobe characteristics and posterior cortical presentations were not associated with *APOE4* status. In addition, no association was found between reduced age of onset and *APOE4* status.
Lehtovirta et al. [53]	58 probable AD patients and 16 controls were evaluated for the effects of age (< 65 or ≥ 65) and disease type (sporadic or familial) on cognitive decline across various measures.	Mean age of *APOE4*+ homozygotes was 66 (*n* = 13; 45% female), *APOE4*+ heterozygotes was 72 (*n* = 24; 46% female), *APOE4*− was 70 (*n* = 21; 52% female), and control group was 72 (*n* = 34; 58% female). No ancestry information was provided.	*APOE4*+ AD patients demonstrated greater amnestic deficits (immediate and delayed recall) with increasing allele load, and earlier age of onset compared to *APOE4*− AD patients.
Weintraub et al. [59]	The *APOE* genotype of 42 patients with primary progressive aphasia (PPA) and AD pathology (PPA/AD) was compared with 1418 patients with autopsy-confirmed AD and amnestic dementia of the Alzheimer type (DAT/AD).	Mean age of symptom onset for PPA/AD was 60.9 (42.9% *APOE4+*, 38.1% female) and DAT/AD was 68.2 (65.7% *APOE4*+, 45.8% female). No ancestry information was provided.	DAT/AD patients were found to be enriched for the *APOE4* allele, while PPA/AD patients were not.
**Lack of** ***APOE4*** **associated with more pronounced non-memory deficits (only)**			
Scheltens et al. [48]	1982 probable AD patients across four large cohorts (Amsterdam Dementia Cohort, ADNI, German Dementia Competence Network, and UCSF Memory and Aging Center) were clustered using neuropsychological data and assigned to either a memory or a non-memory group.	Mean age was 71 (64% *APOE4*+, 54% female). No ancestry information was provided.	Across cohorts, AD patients in the non-memory clusters were less often *APOE4* carriers and had less severe hippocampal atrophy and more severe posterior cortex atrophy compared to the memory group.
Smits et al. [54]	199 probable AD patients (Amsterdam Dementia Cohort) were evaluated using a neuropsychological battery to measure the effects of age of onset (≤ 65 years old or > 65 years old) and *APOE4* status on cognitive decline.	Mean age of *APOE4*+ AD patients was 65 (46% female) and *APOE4*− AD patients was 65 (54% female). No ancestry information was provided.	*APOE4*− AD patients declined faster on language compared to *APOE4*+ AD patients. When taking age into account, early-onset *APOE4*− AD patients declined faster on language, attention, executive control, and visuospatial functioning compared to late-onset *APOE4*+ AD patients. There was no significant difference in decline on memory between groups.
Davidson et al. [55]	627 mild/moderate AD patients were evaluated using cognitive screening tools including the MMSE and the Dementia Rating Scale-2 (DRS-2) to identify cognitive subgroups using latent class analysis.	Mean age was 63.4 for males and 63.8 for females (52% of total subjects were female). All participants were Caucasian.	Four classes were generated (Mild, Attention/Construction, Severe, Memory). The Mild class was the most likely to include *APOE4*+ AD patients, while the Attention/Construction class was least likely to include *APOE4*+ AD patients.
Schott et al. [56]	39 AD patients were assessed using the MMSE, neuropsychological tests, and MRI imaging to investigate *APOE4* frequency in the so-called biparietal AD, characterized as having “combinations of dyscalculia, dyspraxia, visuoperceptual, visuospatial, and spelling deficits with relatively spared memory.”	Mean age of the 10 “biparietal” AD patients was 56.1 (60% female). No ancestry information was provided.	10 “biparietal AD” patients were identified and were found more likely to be *APOE4* non-carriers.
Hashimoto et al. [57]	138 probable AD patients were evaluated for cognitive abilities and regional brain volume using MRI-based techniques.	Mean age of all three groups, *APOE3/3*, *APOE3/4*, and *APOE4/4*, was 69 (*n* = 46; 65% female for each group). Participants were of Japanese ancestry.	No significant effects of *APOE4* status were found on memory function, but there was an association between *APOE4*− AD patients and impairment on WMS-R attention/concentration subtests. Further, *APOE4*+ AD patients demonstrated increased WAIS-R performance and verbal IQ with increasing allele load compared to *APOE4*− AD patients.
***APOE4*** **associated with more pronounced memory deficits** ***and*** **lack of** ***APOE4*** **associated with more pronounced non-memory deficits**			
Wolk et al. [51]	91 mild AD patients (ADNI cohort) were evaluated for phenotypic differences in cognition and regional cortical volume.	Mean age for *APOE4*+ AD patients was 74 (*n* = 67; 43% female) and *APOE4*− AD patients was 74 (*n* = 24; 45% female). No ancestry information was provided.	*APOE4*+ AD patients demonstrated greater impairment on measures of memory retention, whereas *APOE4*− AD patients were more impaired on tests of working memory, executive control, and lexical access.
van der Vlies et al. [52]	229 probable AD patients were assessed for impairment in specific cognitive domains in relation to *APOE4* status using numerous cognitive screening tools.	Mean age of *APOE4* homozygotes was 66 (*n* = 32; 65% female), *APOE4* heterozygotes was 66 (*n* = 132; 56% female), and *APOE4*− was 67 (*n* = 65; 51% female). No ancestry information was provided.	*APOE4*+ AD patients demonstrated greater overall amnestic deficits, while *APOE4*− AD patients were more impaired in domains of naming, executive function, and mental speed.
Kim et al. [58]	846 AD patients and 815 controls were divided into groups based on age (< 65, 65–74, ≥ 75 years) to evaluate regional brain volume and cognitive function in relation to *APOE* genotype.	Mean age for the < 65 group was 58.1 (*n* = 184; 64% female), the 65–74 group was 66.4 (*n* = 252; 67% female), and the ≥ 75 group was 80.3 (*n* = 410; 70% female). Participants were of Korean ancestry.	*APOE4*− AD patients under 75 years old and *APOE3/4* AD patients under 75 years old performed worse on measures of language, visuospatial, and frontal function compared to *APOE4*/*4* AD patients, while *APOE4/4* AD patients over 75 years old performed worse on measures of memory compared to *APOE4*− AD patients.

Although these studies utilized different methodological approaches, the results were generally consistent. For example, Scheltens et al. combined four large probable AD cohorts using a neuropsychologically derived cluster analysis and found two distinct groups—a memory-impaired group and a non-memory-impaired group, with the non-memory-impaired group comprised primarily of younger, *APOE4−* AD patients, as compared to the memory-impaired group [48]. Kim et al. recruited 846 South Korean patients diagnosed with probable AD and categorized them into three groups with respect to their age (< 65, 65–74, and ≥ 75 years old). The authors discovered that younger (< 65 years old) *APOE4−* AD patients performed worse on executive function tasks compared to younger *APOE4*+ AD patients, while intermediate (65–74 year-old) *APOE3/4* AD patients performed worse on visuospatial tasks compared to intermediate *APOE4/4* AD patients, and older (≥ 75 years old) *APOE4/4* AD patients performed worse on verbal memory compared to older *APOE4−* AD patients [58]. Finally, Wolk et al. compared cognitive differences in 67 *APOE4+* vs. 24 *APOE4−* patients diagnosed with mild AD and possessing CSF biomarker profiles consistent with AD [51]. *APOE4+* AD patients performed worse on memory retention, while *APOE4−* AD patients were more impaired on tests of working memory, executive function, and lexical access, but not on confrontational naming.

These results suggest that AD patients likely diverge in their cognitive presentations based on their *APOE* genotype, with *APOE4+* AD patients presenting with relatively more pronounced amnestic deficits than *APOE4−* AD patients, and *APOE4−* AD patients presenting with relatively more non-memory deficits than *APOE4+* AD patients. This conclusion is also consistent with the reported observation that AD patients presenting with atypical phenotypes, such as CBS, fvAD, lvPPA, and PCA, are less likely to be *APOE4* carriers [60, 61].

Interestingly, possession of the *APOE4* allele has also been associated with decreased memory performance in non-demented elderly individuals [62–65], as well as with increased incidence of amnestic MCI vs. non-amnestic MCI [66, 67]. This suggests that possession of the *APOE4* allele may confer increased memory deficits throughout the aging to AD continuum, although it should be noted that *APOE4* carriers have also been found to be at an increased risk of developing several non-AD dementias, including vascular dementia (VaD) [68–71], Lewy body dementia (LBD) [72–74], and frontotemporal dementia (FTD) [75, 76], which often do not present with a predominantly amnestic phenotype.

### *APOE4*+ AD patients have more atrophy in the medial temporal lobe than *APOE4−* AD patients

The cognitive deficits observed in AD patients are a direct result of the pathological abnormalities that occur in a patient’s brain during the course of the disease. AD pathology is characterized by the hallmark accumulation of Aß-containing amyloid plaques and hyperphosphorylated tau-containing neurofibrillary tangles (NFTs). Amyloid plaques are extracellular and accumulate in the brain in a rather diffuse manner, typically starting in the neocortex (Thal phase 1), followed by the entorhinal cortex, hippocampus, and insular cortex (Thal phase 2), and eventually accumulating in subcortical regions such as the basal forebrain and brainstem (Thal phases 3–5) [77]. On the other hand, NFTs are intracellular and accumulate in the brain in a more localized and regionally conserved manner, typically occurring first in the transentorhinal and entorhinal cortex regions (Braak stages I–II), followed by the hippocampus and neighboring neocortical regions (Braak stages III–IV), and eventually accumulating throughout the remainder of the neocortex (Braak stages V–IV) [78]. The third major pathological feature of AD is “brain atrophy,” as measured by volumetric reduction or cortical thinning observed during magnetic resonance imaging (MRI). In general, the atrophy observed in the brains of AD patients has been found to follow along the same regional path as NFTs, with the first signs of volumetric loss observed in the medial temporal lobe during the MCI phase, followed by the neocortical portions of the temporal lobe, then the parietal lobe, and finally the frontal lobe during the course of MCI and AD progression [79]. In addition to these three distinctive features of AD pathology, other important pathological events that also occur during the course of the disease include neuroinflammation, deficits in cellular metabolism, cholinergic dysfunction, aberrant network activity, and cerebrovascular pathology [80].

With respect to *APOE* genotype effects on AD pathology, the most compelling results published to date describe the differing regional patterns of brain atrophy observed in *APOE4*+ vs. *APOE4−* AD patients. While a couple of studies have not observed any differences in brain volume or cortical thickness between *APOE4*+ vs. *APOE4−* AD patients [81, 82], the vast majority of the studies that have investigated this topic to date have found that *APOE4*+ AD patients possess greater volumetric loss or cortical thinning in the medial temporal lobe than *APOE4−* AD patients [51, 57, 58, 83–89], with many reporting that *APOE4*+ vs. *APOE4−* AD patients display volumetric decreases in specific medial temporal lobe structures, such as the hippocampus [57, 83, 86, 87, 89], the amygdala [57, 83, 86, 87], and the entorhinal cortex [84, 89]. Furthermore, many of these studies also reported that *APOE4−* AD patients possess greater volumetric loss or cortical thinning in their frontal and parietal lobes than *APOE4+* AD patients [51, 58, 85, 89, 90] (Table 3).

**Table 3:** Studies investigating the effects of *APOE4* on brain atrophy in AD patients

Study	Study details	Participant details	Study results
**No relationship between brain atrophy and** ***APOE4*** **status**			
Drzezga et al. [81]	32 moderate AD patients matched by demographics and level of cognitive impairment were evaluated for brain volume using cranial MRI and voxel-based morphometry (VBM).	Mean age for *APOE4*+ AD patients was 67 (*n* = 18; 50% female) and *APOE4*− AD patients was 68 (*n* = 14; 35% female). No ancestry information was provided.	Comparisons between *APOE4+* vs. *APOE4*− AD patients showed similar levels and patterns of brain atrophy.
Jack et al. [82]	62 probable AD patients and 125 controls were evaluated for hippocampal volume using MRI.	Mean age for both the *APOE4+* (*n* = 36) and *APOE4*− (*n* = 26) AD patients was 75, while *APOE4*+ control group (*n* = 30) was 80, and *APOE4*− control group (*n* = 95) was 78. No ancestry information was provided.	Although the authors noted that both the AD and control groups trended towards an *APOE4* effect, there were no significant differences in hippocampal volume between *APOE4+* vs. *APOE4*− AD patients
***APOE4*** **associated with increased brain atrophy in the medial temporal lobe**			
Wolk et al. [51]	91 mild AD cases (ADNI cohort) were evaluated for cortical volume using MRI morphometric measures.	Mean age for *APOE4*+ AD patients was 74 (*n* = 67; 43% female) and *APOE4*− AD patients was 74 (*n* = 24; 45% female). No ancestry information was provided.	*APOE4*+ AD patients demonstrated greater brain atrophy in the medial temporal lobe, but less fronto-parietal atrophy compared to *APOE*4− AD patients.
Hashimoto et al. [57]	138 probable AD patients were evaluated for regional brain volume in the hippocampal formation, amygdaloid complex, and whole brain using MRI-based volumetry techniques.	Mean age of all three groups, *APOE3/3*, *APOE3/4*, and *APOE4/4*, was 69 (*n* = 46; 65% female for each group). Participants were of Japanese ancestry.	AD patients demonstrated greater atrophy in the hippocampus and amygdala with increasing *APOE4* alleles, whereas whole brain volume increased with increasing *APOE4* alleles.
Kim et al. [58]	846 AD patients and 815 controls were divided into groups based on age (< 65, 65–74, ≥ 75 years old) to evaluate regional brain volume using MRI.	Mean age for the < 65 group was 58.1 (*n* = 184; 64% female), the 65–74 group was 66.4 (*n* = 252; 67% female), and the ≥ 75 group was 80.3 (*n* = 410; 70% female). Participants were of Korean ancestry.	In total AD patients, a higher number of *APOE4* alleles were associated with cortical thinning in the bilateral medial temporal areas. Moreover, older (≥ 75 years old) *APOE4*+ AD patients had the most severe medial temporal atrophy, while young (< 65 years old) *APOE4*− AD patients had more severe frontal and perisylvian atrophy.
Mattsson et al. [90]	65 Aß-positive AD patients (BioFINDER cohort) were evaluated for tau load and cortical thickness using ^18^F-AV-1451 PET and MRI, respectively.	Mean age for *APOE4*+, AD patients was 72.4 (*n* = 46; 61% female) and *APOE4*− AD patients was 70.1 (*n* = 19; 53% female). No ancestry information was provided.	*APOE4*− AD patients demonstrated reduced thickness in the lateral and parietal areas compared to APOE4+ AD patients.
Filippini et al. [83]	83 AD cases were evaluated for regionally specific brain cortical volume using voxel-based morphometry (VBM).	Mean age of *APOE4*+ homozygotes was 75.5 (*n* = 15; 80% female), *APOE4*+ heterozygotes was 81.1 (*n* = 39; 53% female), and *APOE4*− was 75.8 (*n* = 29; 48% female). No ancestry information was provided.	Bilateral medial and anterior temporal lobes, including amygdala, hippocampal, and entorhinal cortex, and orbitofrontal gray matter volume decreased with increasing *APOE4* allele load.
Juottonen et al. [84]	27 probable AD patients and 31 controls were evaluated for entorhinal cortex volume using MRI.	Mean age of *APOE4*+ AD patients was 70 (*n* = 16; 37% female), *APOE4*− AD patients was 69 (*n* = 11; 54% female), and control was 72 (*n* = 31; 64% female). No ancestry information was provided.	*APOE4*+ AD patients demonstrated greater atrophy in the entorhinal cortex, compared to *APOE4*− AD patients, with only the left entorhinal cortex reaching statistical significance.
Pievani et al. [85]	29 AD patients and 29 age- and sex-matched controls were evaluated for cortical volume using MRI.	Mean age of *APOE4*+ AD patients was 71 (*n* = 15; 93% female), *APOE4*− AD patients was 68 (*n* = 14; 50% female), and control was 69 (*n* = 29; 72% female). No ancestry information was provided.	*APOE4*+ AD patients demonstrated greater brain atrophy in the temporal cortex, right occipital pole, and, to a lesser less degree, in the posterior cingulate, left orbitofrontal and dorsal fronto-parietal cortex compared to *APOE4*− AD patients.
Lehtovirta et al. [86]	58 probable AD patients and 34 age- and sex-matched controls were evaluated for hippocampal, amygdala, and frontal lobe volume, as well as cerebral blood flow, using MRI and SPECT, respectively.	Mean age of *APOE4*+ homozygotes was 66 (*n* = 13; 45% female), *APOE4*+ heterozygotes was 72 (*n* = 24; 46% female), *APOE4*− was 70 (*n* = 21; 52% female), and control group was 72 (*n* = 34; 58% female). No ancestry information was provided.	*APOE4*+ homozygous AD patients demonstrated greater brain atrophy in the medial temporal structures, hippocampus, and amygdala. However, the frontal lobe volume did not significantly differ between groups.
Lehtovirta et al. [87]	26 probable AD cases and 16 age- and sex-matched controls were evaluated for hippocampal, amygdala, and frontal lobe volume using MRI.	Mean age of *APOE4/4* AD patients was 65 (*n* = 5; 60% female), APOE3/4 AD patients was 71 (*n* = 9; 44% female), *APOE4*− (APOE2/3 and APOE3/3) AD patients was 68 (*n* = 12; 41% female), while control was 70 (*n* = 16; 62% female). No ancestry information was provided.	*APOE4/4* AD patients had the most prominent brain atrophy in the hippocampus and amygdala, and differed significantly from *APOE3/4* and *APOE4*− AD patients in the volume of the right hippocampus and right amygdala. There were no significant differences between groups in the frontal lobe.
Tanaka et al. [88]	34 probable AD patients and 22 controls were evaluated for morphological and functional changes using CT, MRI, and SPECT.	Mean age of *APOE4/4* AD patients was 80.8 (*n* = 4), APOE3/4 AD patients was 81 (*n* = 8), *APOE4*− (APOE3/3) AD patients was 84.6 (*n* = 22), while control was 82 (*n* = 22). No gender information was provided. Participants were of Japanese ancestry.	*APOE4* allele dose did not affect overall brain volume during the course of the disease. However, the inferior temporal and infero-medial temporal areas were statistically lower in volume in *APOE4*+ AD patients, while the temporal horn was higher in volume in *APOE4*+ vs. *APOE4*− AD patients.
Geroldi et al. [89]	28 mild to moderate AD patients and 30 controls were evaluated for hippocampal, entorhinal cortex, anterior temporal, and frontal lobe volume using MRI.	Mean age for the AD patients was 73 (*n* = 28; 78% female) while control was 69 (*n* = 30; 67% female). No ancestry information was provided.	There was increasing atrophy in the hippocampus, entorhinal cortex, and anterior temporal lobes with increasing *APOE4* dose. In contrast, larger volumes of the frontal lobes were observed with increasing *APOE4* dose.

Importantly, a number of these studies noted a direct correlation between the regional brain atrophy patterns that they observed between *APOE4*+ vs. *APOE4−* AD patients and the differences in cognitive profile that they observed in these same patients [51, 57, 58, 84, 87, 90]. For example, in the Scheltens et al. study, the authors also analyzed MRI data from their four large probable AD cohorts and observed that in their non-memory-impaired group, which was enriched for *APOE4−* AD patients, there was less hippocampal volume loss and more posterior cortex volume loss than in the memory-impaired group [48]. And in the Kim et al. study, the authors measured cortical thinning using MRI in their 846 South Korean probable AD patients and found that in the younger (< 65 years old) *APOE4−* AD patients, who performed worse on executive function tasks, there was increased bilateral cortical thinning in their lateral frontal, medial frontal, and perisylvian areas compared to the younger *APOE4−* AD patients, whereas in the older (≥ 75 years old) *APOE4*+ AD patients, who performed worse on verbal memory tasks, there was increased bilateral cortical thinning in their medial temporal areas compared to the older *APOE4−* AD patients [58]. Finally, in the Wolk et al. study, the authors used MRI to measure brain volume and cortical thickness in their mild AD patients and found that *APOE4+* AD patients, who performed worse on memory retention, displayed greater hippocampal volume loss than *APOE4−* AD patients, whereas *APOE4−* AD patients, who performed worse on working memory, executive function, and lexical access, displayed decreased cortical thickness in their superior parietal lobule, precuneus, and angular gyrus than *APOE4+* AD patients.

### *APOE4*+ AD patients do not appear to have higher Aß levels than *APOE4−* AD patients

As noted above, the regional brain atrophy that is observed in AD patients is thought to be a direct result of the tau accumulation that progressively occurs in neurons within these brain regions. And it is believed that this tau accumulation and the regional progression of NFTs likely occur downstream of the Aß accumulation/amyloid plaque deposition that begins early in AD pathogenesis. Given this information, it is important to determine whether the presentation of these two hallmark pathologies also displays heterogeneity in *APOE4*+ vs. *APOE4−* AD patients, and how this presentation may relate to the differences in brain atrophy and cognitive deficits that are observed in these patients. In terms of Aß, it has been well documented that individuals who carry the *APOE4* allele accumulate Aß in their brains at an earlier age than non-carriers, and that this occurs long before the onset of AD. For example, a 2015 meta-analysis by Jansen et al. revealed that by the time *APOE4/4* carriers turn 40 years old, about 15% of them will already be positive for cerebral Aß (as detected by PET or CSF), whereas this threshold is not reached until 55 years of age for *APOE3/4* carriers and 65 years of age for *APOE3/3* carriers [91]. However, Aß levels have been shown to plateau before the clinical diagnosis of AD [92], so any differences in Aß levels associated with *APOE* genotype are not expected to be as dramatic once a patient converts to AD as it is during the linear phase of Aß accumulation. For this reason, it is perhaps not surprising that the handful of studies that have compared the levels of Aß in *APOE4*+ vs. *APOE4−* AD patients have shown conflicting results, with some studies reporting increased Aß levels in the brains of *APOE4*+ AD patients compared to *APOE4−* AD patients [81, 93, 94], some reporting no changes in Aß levels between these two groups [95, 96], and some reporting decreased Aß levels in the brains of *APOE4*+ AD patients compared to *APOE4−* AD patients [97, 98] (Table 4).

**Table 4:** Studies investigating the effects of *APOE4* on amyloid plaques in AD patients

Study	Study details	Participant details	Study results
***APOE4*** **associated with increased amyloid plaques deposition**			
Tiraboschi et al. [93]	296 AD autopsy cases were evaluated for amyloid plaques and NFTs in the hippocampus, and midfrontal, inferior parietal, and superior temporal cortices.	Mean age at death of *APOE4*+ homozygotes was 76.4 (*n* = 38; 55% female), *APOE*+ heterozygotes was 80.1 (*n* = 149; 54% female), and *APOE4*− was 80.2 (*n* = 109; 58% female). No ancestry information was provided.	*APOE4/4* AD patients demonstrated significantly more amyloid plaques and NFTs in neocortical regions than *APOE3/4* or *APOE4*− AD patients.
Drzezga et al. [81]	32 moderate AD patients matched for demographic and cognitive impairment were evaluated for amyloid plaque deposition via PIB-PET imaging.	Mean age for *APOE4*+ AD patients was 67 (*n* = 18; 50% female) and *APOE4*− AD patients was 68 (*n* = 14; 35% female). No ancestry information was provided.	*APOE4*+ AD patients exhibited significantly higher and more extended amyloid plaque deposition, especially in bilateral prefrontal and temporoparietal cortex compared to *APOE4*− AD patients.
Berg et al. [94]	186 AD autopsy cases and 13 controls were evaluated for multiple brain histological markers of AD, including brain densities of amyloid plaques and NFTs.	Broken down by CDR, the mean age at death of CDR = 0 was 82.4 (*n* = 13; 38% female), CDR = 0.5 was 88.6 (*n* = 17; 52% female), CDR = 1 was 87.8 (*n* = 8; 50% female), CDR = 2 was 81.2 (*n* = 17; 52% female), and CDR = 3 was 79.8 (*n* = 144; 55% female). No ancestry information was provided.	Controlling for dementia severity, plaque densities were weakly associated with *APOE4* status in the hippocampus. The degree of CAA was more strongly associated with *APOE4* status.
**No relationship between amyloid plaque deposition and** ***APOE4*** **status**			
Rowe et al. [95]	53 mild AD, 57 MCI, and 177 control cases (AIBL cohort) were evaluated for amyloid plaque deposition via PIB-PET imaging.	Mean age of AD patients was 72.6 (*n* = 53; 56% female), MCI patients was 75.5 (*n* = 57; 49 female), and controls was 71.6 (*n* = 177; 49% female). No ancestry information was provided.	*APOE4*+ MCI patients and controls exhibited statistically higher PIB binding than *APOE4*− MCI patients and controls. However, there were no differences observed between *APOE4+* vs. *APOE4*− AD patients*.*
Landen et al. [96]	44 AD, 11 vascular dementia, and 29 age-matched control autopsy cases were evaluated for amyloid plaques and NFTs in the hippocampus and frontal cortex.	Mean age at death for *APOE4*+ AD patients was 78.1 (*n* = 32), *APOE4*− AD patients was 82.5 (*n* = 12), *APOE4*+ VaD patients was 76.7 (*n* = 3), *APOE4*− VaD patients was 80.1 (*n* = 8), and *APOE4*+ controls was 71.0 (*n* = 19), *APOE4*− controls was 75.7 (*n* = 10). AD patients were 61% female, VaD patients were 27% female, and controls were 34% female. No ancestry information was provided.	No association was found between *APOE4* status and amyloid plaque or NFT levels in either the AD, vascular dementia, or control groups.
***APOE4*** **associated with decreased amyloid plaque deposition**			
Ossenkoppele et al. [97]	22 *APOE4*− AD patients, 40 *APOE3/4* AD patients, and 22 *APOE4*/4 AD patients were evaluated for amyloid plaques and brain metabolism using PIB-PET and FDG-PET, respectively.	Mean age of *APOE4*+ homozygotes was 65 (*n* = 22; 41% female), *APOE4*+ heterozygotes was 62 (*n* = 40; 38% female), and *APOE4*− was 61 (*n* = 22; 27% female). No ancestry information was provided.	*APOE4*− AD patients exhibited increased PIB binding in the frontal cortex compared to *APOE4+* AD patients, while *APOE4*− AD patients had less profound metabolic impairment in the posterior parts of the cortex compared to *APOE4+* AD patients.
Lehmann et al. [98]	52 probable AD and 52 control cases were evaluated for amyloid plaque deposition and brain metabolism using PIB-PET and FDG-PET, respectively.	Mean age for *APOE4*+ AD patients was 64.3 (*n* = 23; 48% female), *APOE4*− AD patients was 62.7 (*n* = 29; 41% female), and controls was 72.3 (*n* = 52; 58% female). No ancestry information was provided.	*APOE4*− AD patients exhibited increased global amyloid plaque burden compared to matched *APOE4+* AD patients. In contrast, *APOE4+* AD patients exhibited greater medial temporal hypometabolism compared to *APOE4*− AD patients.

Looking closely at these studies, it is difficult to make a conclusive statement about how exactly *APOE* genotype affects Aß levels or amyloid plaque distribution in the brains of AD patients. For example, the studies by Drzezga et al. (32 patients with moderate AD) [81], Rowe et al. (53 patients with mild AD) [95], and Lehmann et al. (52 patients with probable AD) [98] each utilized Pittsburgh Compound B (PIB) PET analysis on age- and cognition-matched AD patients who were confirmed to be Aß-positive, but with each study arriving at a different conclusion about the relative levels of Aß in *APOE4*+ vs. *APOE4−* AD patients. Perhaps future work on this topic will reveal more regionally specific differences in how Aß is distributed in the brains of *APOE4*+ vs. *APOE4−* AD patients. This is hinted at by the Lehmann et al. study, where the observed decrease in Aß in *APOE4*+ AD patients was primarily localized to the right lateral frontotemporal regions of the brain [98].

Of course, it is also important to note that amyloid plaques are only one manifestation of Aß pathology that can occur in the brain. Aß can also build up in the walls of arteries (cerebral amyloid angiopathy; CAA) or inside of neurons (intraneuronal Aß). Interestingly, several studies have reported that *APOE4*+ AD patients have a more frequent CAA comorbidity than *APOE4−* AD patients [94, 99–101]. As for intraneuronal Aß, while one study did report that post-mortem brains from *APOE4*+ AD patients possess higher levels of intraneuronal Aß than those from *APOE4−* AD patients [102], much more investigation is required before any conclusive statements can be made on this topic.

### *APOE4*+ AD patients appear to develop more tau pathology in their medial temporal lobe than *APOE4−* AD patients

As with Aß, there have been numerous reports that *APOE4* carriers possess higher levels of tau pathology than non-carriers *prior* to AD onset, although this effect on preclinical tau pathology does not seem to be nearly as robust as it is with *APOE4*’s effects on preclinical Aß levels. For example, in a study where Braak and colleagues analyzed autopsied brain tissues specifically from individuals who reached Braak stage I (transentorhinal cortex only) at a relatively young age (less than 47 years old), the authors reported a significant increase in the percentage of *APOE4* carriers in this group (36%) vs. the percentage of *APOE4* carriers in the control group (16%) [103]. A later, more generalized autopsy study from Braak and colleagues also observed that women who were *APOE4* carriers met the criteria for Braak stages II (entorhinal cortex) and III (hippocampus) 3 years earlier than non-carriers [104]. Several more recent studies have also reported a female-dominant effect of *APOE* genotype on tau levels prior to AD diagnosis [12, 105, 106]. In each of these studies, the authors reported that *APOE4* possession increases CSF tau levels specifically in female *APOE4* carriers, with two of the studies reporting that this *APOE4*-associated effect on CSF tau levels was only present when the women were positive for Aß pathology [105, 106].

In regard to *APOE4*+ vs. *APOE4−* AD patients, tau pathology also appears to differ according to *APOE* genotype, although the primary differences here appear to revolve around the regional pattern of NFT distribution, as opposed to the overall levels (Table 5). For example, Murray et al. have reported that, when AD autopsy cases were divided into three distinct groups based on the regional pattern of the NFT pathology observed (“hippocampal-sparing,” “typical,” and “limbic predominant”), there was a trend towards fewer *APOE4* carriers in the “hippocampal-sparing” AD group, and there were significantly more late-onset (greater than 65 years old at diagnosis) *APOE4* carriers vs. non-carriers in the “limbic predominant” AD group [61]. Although a more recent study failed to replicate this finding in a set of AD autopsy cases enriched for atypical presentation (in which *APOE4* carriers were underrepresented), there did appear to be a trend (*p* = 0.0992) towards more *APOE4* carriers among “limbic predominant” AD cases and fewer *APOE4* carriers among “hippocampal-sparing” AD cases [107]. Interestingly, a recent follow-up paper by Murray and colleagues also reported that *APOE4*+ “typical” AD patients, as compared to *APOE4−* “typical” AD patients, possess more NFT pathology in their nucleus basalis of Meynert (nbM), the major source of cholinergic innervation in the brain [110].

**Table 5:** Studies investigating the effects of *APOE4* on neurofibrillary tangles in AD patients

Study	Study details	Participant details	Study results
**No relationship between NFT deposition/distribution and** ***APOE4*** **status**			
Petersen et al. [107]	94 AD autopsy cases enriched for atypical AD presentation were evaluated for patterns of regional NFT accumulation in six selected neocortical and hippocampal regions.	Age range at death for the entire group was 51–73 at age of onset and 63–86 at death (*n* = 94; 40% female). No ancestry information was provided.	No significant difference in regional NFT density was found between *APOE4+* vs. *APOE4*− AD patients, although there was a trend (*p* = 0.0992) towards more *APOE4*− AD patients in the hippocampal-sparing group and more *APOE*+ AD patients in the limbic predominant group.
***APOE4*** **associated with increased NFT deposition in the medial temporal lobe**			
Murray et al. [61]	889 AD autopsy cases were used to study regional density and distribution of NFTs. Cases were classified as hippocampal-sparing, typical, or limbic predominant based on their relative NFT distribution.	Average age at death for the hippocampal-sparing subtype was 73 (*n* = 97; 37% female), typical subtype was 79 (*n* = 665; 55% female), and limbic predominant subtype was 86 (*n* = 127; 69% female). No ancestry information was provided.	Significantly more *APOE4*+ AD patients were included in the late-onset (> 65 years old) limbic predominant group, while a trend towards fewer *APOE4*+ AD patients were included in the hippocampal-sparing group.
Ossenkoppele et al. [108]	20 cases with either MCI or probable AD, and 15 Aß-negative cognitively normal individuals were evaluated for ^18^F-AV-1451 tau PET ligand uptake, as well as PIB-PET and FDG-PET.	Mean age for PCA patients was 63 (*n* = 7; 42% female), lvPPA patients was 65 (*n* = 5; 80% female), amnestic AD patients was 67 (*n* = 5; 40% female), non-amnestic AD patients was 59 (*n* = 1; 0% female), behavioral/dysexecutive variant AD patients was 59 (*n* = 1; 0% female), and CBS patients was 60 (*n* = 1, 100% female). No ancestry information was provided.	*APOE4+* AD patients exhibited increased ^18^F-AV-1451 uptake in the bilateral medial temporal and right temporoparietal cortex compared to *APOE4*− AD patients.
Whitwell et al. [109]	62 Aß-positive AD patients were evaluated for ^18^F-AV-1451 tau PET ligand uptake in the entorhinal cortex (EC) relative to whole cortex (C). Using *K*-median cluster analysis, cases were classified into three categories: EC^Lo^/C^Lo^, EC^Lo^/C^Hi^, and EC^Hi^/C^Hi^.	Mean age for the EC^Lo^/C^Lo^ group was 76 (*n* = 21; 38% female), the EC^Lo^/C^Hi^ group was 64 (*n* = 21; 57% female), and the EC^Hi^/C^Hi^ group was 62 (*n* = 20; 65% female). No ancestry information was provided.	*APOE4* frequency was found to be significantly lower in the EC^Lo^/C^Hi^ group (48%) relative to the EC^Lo^/C^Lo^ (84%) and EC^Hi^/C^Hi^ (74%) groups. Thus, in the context of high cortical tau load (but not low cortical tau load), fewer *APOE4*+ AD patients possessed low tau load in the entorhinal cortex compared to *APOE4*− AD patients.
Mattsson et al. [90]	65 Aß-positive patients with either MCI or AD (BioFINDER cohort) were evaluated for ^18^F-AV-1451 tau PET ligand uptake and cortical thickness via MRI.	Mean age for *APOE4*+ AD patients was 72.4 (*n* = 46; 61% female) and *APOE4*− AD patients was 70.1 (*n* = 19, 53% female). No ancestry information was provided.	*APOE4+* AD patients exhibited increased tau load in the entorhinal cortex relative to the whole cortex, and lower NFT load in the lateral parietal, medial parietal, occipital, and whole brain cortical areas compared to *APOE4*− AD patients.
***APOE4*** **associated with increased NFT deposition in other brain regions**			
Al-Shaikh et al. [110]	1361 AD subtypes and 103 controls (FLAME cohort) were assessed for NFT accumulation and neuronal density differences between different AD subtypes (hippocampal-sparing, typical, or limbic predominant).	Mean age at death for the hippocampal-sparing subtype was 72 (*n* = 175; % female), the limbic predominant subtype was 86 (*n* = 172; 70% female), the typical subtype was 81 (*n* = 1014; 54% female), and controls was 73 (*n* = 103; 46% female). No ancestry information was provided.	Within the “typical” AD group, *APOE4+* AD patients exhibited higher NFT pathology in their nucleus basalis of Meynert (nbM), located in the basal forebrain, compared to *APOE4*− AD patients.

To interrogate this correlation between *APOE* genotype and tau pathology in living individuals, researchers have begun utilizing recently developed tau PET imaging ligands to compare tau levels in *APOE4*+ vs. *APOE4−* AD patients. For example, a small study by Ossenkoppele et al. utilizing the ^18^F-AV-1451 tau PET ligand in 20 individuals diagnosed with MCI or AD found increased uptake of the PET ligand in bilateral medial temporal and right temporoparietal cortex of *APOE4*+ patients, as compared to *APOE4−* patients [108]. And in a study by Whitwell et al. employing ^18^F-AV-1451 to investigate tau deposition in 62 amyloid-positive AD patients with a mix of typical and atypical AD presentations, the authors separated their subjects into three groups (EC^Lo^/C^Lo^, EC^Lo^/C^Hi^, and EC^Hi^/C^Hi^) based on the amount of tau deposition they observed in the entorhinal cortex (EC), as compared to the whole cortex (C) [109]. The authors found that the *APOE4* frequency was significantly lower in the EC^Lo^/C^Hi^ group, suggesting that *APOE4−* AD patients have less relative tau accumulation in the entorhinal cortex region than *APOE4+* AD patients in the context of high cortical tau load. Finally, in a study by Mattsson et al. that also utilized the ^18^F-AV-1451 tau PET ligand, again on a mixed group of MCI and AD patients (65 patients total), the authors reported an increased tau load in the entorhinal cortex (relative to the whole cortex) of *APOE4*+ patients compared to *APOE4−* patients, whereas the tau load in the parietal and occipital lobes was higher in *APOE4−* patients compared to *APOE4*+ patients [90].

To be clear, these studies on tau pathology in *APOE4*+ vs. *APOE4−* AD patients are still somewhat preliminary, with additional work required to confidently answer this question. Specifically, additional tau PET imaging ligand studies are required in pure AD populations, and with larger sample sizes. Also, as with the other studies on this topic, analysis of specific subgroupings needs to be performed with respect to age, gender, and ancestral background. However, based on these early results, it does appear that *APOE4*+ AD patients may possess relatively more NFTs in the medial temporal lobe, most notably in the entorhinal cortex, while *APOE4−* AD patients may possess more NFTs in other cortical regions, such as the frontal and parietal lobes.

## Conclusions

### Primary findings and key limitations

The majority of the previous research investigating the relationship between *APOE4* and AD has focused on elucidating the patterns and mechanisms associated with the increased *risk* of developing AD among *APOE4* carriers. And for good reason, after all, *APOE4* is the primary genetic risk factor for sporadic AD. However, the possibility that *APOE4* may also affect the cognitive and pathological presentation of AD deserves significant attention, as this possibility may elucidate differing pathogenic mechanisms between *APOE4*+ vs. *APOE4−* AD patients, both before and after disease onset, and may have important implications for how we should therapeutically treat *APOE4+* vs. *APOE4−* AD patients.

Overall, the studies that have been performed on this topic to date suggest that *APOE4+* vs. *APOE4−* AD patients do appear to possess both cognitive and pathological heterogeneity in their presentation of the disease, as depicted in Fig. 1. Specifically, the neuropsychological studies outlined above show that *APOE4+* AD patients appear to possess relatively more pronounced memory deficits than *APOE4−* AD patients, while *APOE4−* AD patients appear to possess relatively more pronounced non-memory deficits (particularly deficits in executive function, visuospatial abilities, and language) than *APOE4+* AD patients. The literature also points to divergent pathological underpinnings that likely explain the differences in cognitive profiles related to an AD patient’s *APOE* genotype. Most notably, *APOE4+* AD patients appear to possess relatively more brain atrophy in their medial temporal lobe than *APOE4−* AD patients, while *APOE4−* AD patients appear to possess relatively more brain atrophy in their frontal and parietal lobes than *APOE4+* AD patients. The literature also suggests that the upstream trigger of these regional brain atrophy differences is likely to be the observed differences in the regional distribution of NFTs in *APOE4+* vs. *APOE4−* AD patients, with *APOE4+* AD patients possessing a greater relative accumulation of NFTs in their medial temporal lobe (particularly in the entorhinal cortex) than *APOE4−* AD patients, and *APOE4−* AD patients possessing relatively more NFTs in their frontal and parietal lobes than *APOE4+* AD patients. However, due to the limited number of studies performed using recently developed tau PET imaging ligands, this last conclusion is particularly unresolved.

It should also be noted that an AD patient’s *APOE* genotype may affect the presentation of several additional brain pathologies not covered in the “Results” section of this review. For example, in the previously discussed Lehmann et al. study, where the authors observed regional decreases in Aß deposition in *APOE4*+ vs. *APOE4−* AD patients, the authors also reported regional differences in glucose metabolism (as measured by FDG-PET), with *APOE4+* AD patients displaying more hypometabolism in bilateral medial temporal and right lateral temporal regions than *APOE4−* AD patients, while *APOE4−* AD patients displayed more hypometabolism in other cortical areas, including supplementary motor cortex and superior frontal gyrus [98]. Furthermore, autopsied brains from *APOE4*+ vs. *APOE4*−AD patients have also been reported to possess increased levels of two pathological comorbidities commonly associated with AD: TDP-43 [111–113] and Lewy bodies [114, 115].

As noted throughout this review, there are a number of limitations in the studies we cited, which decreases the overall confidence with which we can assert that there is a definitive difference in disease presentation between *APOE4+* vs. *APOE4−* AD patients. For example, some of the studies performed on this topic utilized relatively small sample sizes, which may result in type II ("false-negative") statistical errors. In addition, many of the studies we cited utilized “probable AD” for their AD diagnosis, which may result in type I ("false-positive") statistical errors. Lastly, inherent differences among *APOE4* carriers, like age, gender, and ancestral background, are likely to modulate the effects of *APOE* genotype on AD presentation, a possibility that requires much more investigation.

In order to address these issues, we propose that additional studies comparing the cognitive and pathological presentation of AD in *APOE4+* vs. *APOE4−* AD patients should include the following criteria: (1) Comprehensive neuropsychological testing, or numerous cognitive tests measuring multiple cognitive domains, should be utilized to diagnose AD patients. Cognitive screeners, such as the MMSE, are helpful in identifying individuals who require more comprehensive assessment, but robust neuropsychological tests are far more capable of making accurate diagnoses and clear determinations of the severity of a patient’s cognitive impairment. (2) Pathological diagnosis should be confirmed using established biomarkers such PET tracers or CSF measurements, or histology on post-mortem tissues if the subjects are deceased. (3) Large, diverse cohorts of AD patients should be utilized. These cohorts should include hundreds of participants with different ages, genders, and ancestral backgrounds. Power analysis should be performed not only for the cohort as a whole, but also for the individual demographic subgroupings, in order to allow for statistically significant results from each independent subgroup. (4) All three of the first three criteria should be utilized in tandem in order to carefully match the *APOE4+* AD patients to the *APOE4−* AD patients with which they are being compared. Matched *APOE* genotype groups should possess similar neuropsychological profiles, similar pathology levels, and similar demographics, although variations may be necessary depending on the specific question being tested. We anticipate that these robust future studies will definitively determine whether *APOE4+* vs. *APOE4−* AD patients possess the cognitive and pathological heterogeneity that the initial studies on this topic suggest.

### Why disease heterogeneity is important

In recent years, disease heterogeneity has gained increased attention in AD research, with numerous publications reporting on divergent aspects of AD such as atypical neuropsychological profiles and mixed pathologies in AD patients [116–121]. One reason why the topic of disease heterogeneity is so important in AD research is that it suggests a previously unappreciated complexity that may make therapeutic treatment of AD more difficult (and could also help to explain past clinical trial failures). If AD is not the single, uniform disease that researchers once believed it to be, then a single therapeutic strategy may not be able to help all AD patients equally. In respect to *APOE* genotype, disease heterogeneity may even point to divergent pathological mechanisms that will be particularly important to understand when attempting to treat *APOE4* carriers vs. non-carriers.

On that topic, there have been numerous examples of therapies showing efficacy in *APOE4* carriers, but not in non-carriers, or vice-versa. For example, currently approved acetylcholinesterase inhibitors have often been reported as having differential effects on *APOE4+* vs. *APOE4−* AD patients, although these results have been mixed [122–125]. Investigations of intranasal insulin as an AD treatment have also shown mixed results, with an acute insulin treatment showing memory improvement in *APOE4−* MCI and AD patients, but not *APOE4+* MCI and AD patients [126], while a chronic insulin treatment has shown memory improvement in *APOE4+* MCI and AD patients, but not *APOE4−* MCI and AD patients [127]. Differential *APOE* genotype effects have also been reported for the treatment of mild-to-moderate AD patients using the diabetes drug rosiglitazone, with *APOE4−* AD patients, but not *APOE4+* AD patients, showing cognitive improvement [128]. Furthermore, the retinoid x receptor (RXR) agonist bexarotene has been shown to reduce Aß levels in *APOE4−* AD patients, but not in *APOE4+* AD patients [129]. And in a phase 3 clinical trial of the anti-Aß antibody bapaineuzumab for mild-to-moderate AD, reductions of both Aß and tau levels were observed in *APOE4+* AD patients, but not in *APOE4−* AD patients [130].

Given these potential differences in treatment efficacy for *APOE4+* vs. *APOE4−* AD patients, it is important not only to elucidate any overall cognitive and pathological heterogeneity between these two groups, but also to understand the underlying mechanisms that may drive this heterogeneity. Indeed, the discovery of divergent pathological mechanisms between *APOE4+* vs. *APOE4−* AD patients would not only point to important treatment differences for these two patient groups, but it could also help clarify the mechanism of AD pathogenesis in general. The majority of AD research has focused on Aß and tau accumulation, the pathological hallmarks of the disease. However, understanding the ways in which differential isoform expression of *APOE*, which primarily plays a role in cholesterol and lipid trafficking, mediates AD presentation would add important context to how AD develops and progresses.

### Potential mechanisms

For the most part, the studies referenced in this review do not attempt to pinpoint the underlying mechanism(s) responsible for the heterogeneity that they report between *APOE4+* vs. *APOE4−* AD patients. However, it is worth discussing the potential mechanisms that may be responsible for this heterogeneity. First off, although the pathological differences that occur in *APOE4+* vs. *APOE4−* AD patients appear to center around tau pathology and the resulting brain atrophy, it is still quite possible that the earlier onset of Aß pathology that occurs in *APOE4* carriers vs. non-carriers may play a direct role in the pathological differences that appear to occur in *APOE4+* vs. *APOE4−* AD patients. If tau aggregation is in fact a direct result of Aß pathology, as is proposed by the amyloid cascade hypothesis, it is probable that the earlier increases in Aß accumulation that are observed in the brains of *APOE4* carriers would lead to an early and prolonged accumulation of tau pathology within the entorhinal cortex and hippocampus, the brain regions where NFTs are first observed. This possibility is hinted at by the recent studies showing that women who are positive for both *APOE4* and Aß have higher levels of CSF tau compared to other groups [105, 106], even in the absence of any cognitive decline [106]. That said, it would be anticipated that this increased early accumulation of tau pathology in the medial temporal lobe of *APOE4* carriers would also translate to increased tau pathology in the fronto-parietal lobes as the disease progresses. However, these initial studies have observed the exact opposite, with *decreased* tau pathology and neurodegeneration occurring in the fronto-parietal lobes of *APOE4*+ vs. *APOE4−* AD patients.

Alternatively, it is possible that the mechanisms responsible for this observed cognitive and pathological heterogeneity in *APOE4*+ vs. *APOE4−* AD patients are independent of *APOE4*’s effects on Aß. To that end, it is important to note that *APOE4* expression has been found to have a deleterious effect on numerous Aß-independent pathways within the brain, including cholesterol/lipid metabolism [131–133], endosomal-lysosomal processing [134–142], energy metabolism [143–148], neuroinflammation [149–151], and cerebrovascular integrity [152–155]. Furthermore, *APOE* appears to be highly expressed in the medial temporal lobe compared to other brain regions, as shown in this spatial modeling of *APOE* mRNA levels derived from Allen Brain Atlas mRNA expression data (http://www.meduniwien.ac.at/neuroimaging/lib/dlpage.php?value=348&name=apolipoprotein%20E) [156]. Therefore, cells in the medial temporal lobe of *APOE4* carriers may be particularly susceptible to deficits in the biological pathways listed above.

Clearly, there are still many questions left to be answered with regard to this apparent heterogeneity in *APOE4+* vs. *APOE4−* AD patients, which we anticipate future studies will help to elucidate. We believe that validating and interrogating this *APOE4*-associated heterogeneity will yield important information for how best to treat AD patients based on their specific *APOE* genotype. In addition, uncovering the biological mechanism(s) responsible for this apparent heterogeneity may pave the way for the discovery of novel therapeutic strategies for treating or preventing AD in general.

## Data Availability

Not applicable
